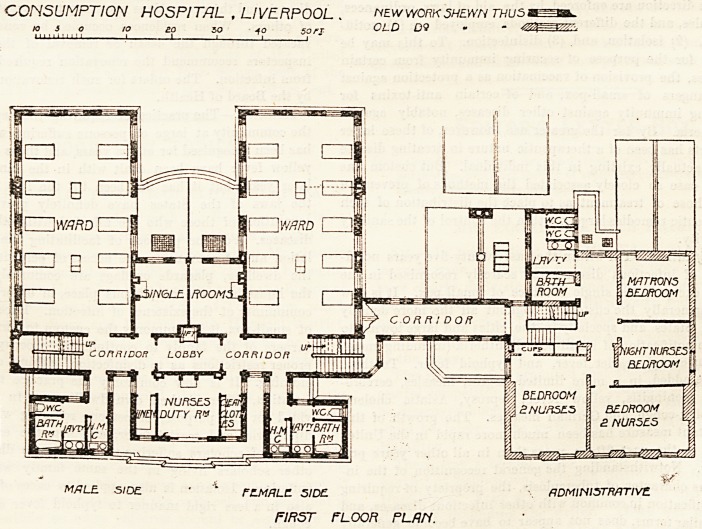# The Liverpool Hospital for Consumptives

**Published:** 1906-01-13

**Authors:** 


### THE LIVERPOOL HOSPITAL FOR CONSUMPTIVES.

This hospital occupies a site at the corner of Rodney Street and Mount Pleasant, and there is lying to the south of it a considerable space walled in as a garden. It is always difficult to criticise a hospital which has been built in a town where land is difficult to get, where there is but little choice of suitable sites, and where committees, medical officers, and architects are all anxious to get as much as they can for the available capital at command. And much depends upon the object the hospital has in view, that is, the class of patients to be received in it. For instance, if this hospital is intended as a temporary residence for consumptives awaiting transfer to a sanatorium in the country, a great deal might be said in its favour; and even if for more permanent residence it may be said to have some good points.

The principal entrance is in Mount Pleasant; and on entering the hall the porter's room is found on the right-hand side, and the doctor's room on the left. Passing through the hall a corridor is reached, and this gives communication with the smoking-room, the dining-hall, and the day rooms of the hospital proper; and a branch corridor gives access to the administrative department which contains the matron's rooms, nurses' sitting-room, servants' hall, kitchen, larder, etc. A considerable part of this wing is an old building which has been added to and altered so as to adapt it to its present purpose. Returning to the patients' block, we find that the dining-hall is wedged in between the two day rooms. There are two windows and a door in its south aspect and the light and ventilation will be increased by the skylights in the roof and to some extent by the doors at the north end; still it can hardly be described as an ideal dining-hall for consumptive patients. The day rooms have a fair number of windows; but the greater part of one side of each room is, of course, blocked by the central position of the dining-hall, and it is, therefore, only at their south ends, or rather at the ends of the east and west walls near the south, that windows are placed exactly opposite each other; while it is just this direct cross ventilation that counts for so much in keeping the air of a room absolutely pure. Still, with the site at disposal, it does not seem easy to suggest any arrangement which would obviate these objections unless it were possible to alter the whole plan. Each day room is provided with a verandah.

The staircases spring from the ends of the main corridor; and on reaching the first-floor landing we find two dormitory wards running from the corridor southwards, and two

GROUND FLOOR PLAN.

FIRST FLOOR PLAN.

single-bedded rooms are placed between these wards, corresponding to the north end of the dining-hall on the ground floor. Each dormitory ward contains ten beds, and is well lighted; but we regret to have to state that one bed in each ward has a window on one side only, and another bed has no window at all; this faulty arrangement being necessary owing to the central position of the single-bedded rooms. The distance between the dormitory blocks seems to be only about twenty-two feet. Part of the space over the dining-hall is utilised as a balcony, and it is divided into two sections, one for men and one for women. The first floor has also a nurses' duty room with ward-kitchen attached, and the sanitary annexes are properly arranged and are cut off from the main corridor by cross-ventilated passages.

The second floor contains eight single-bedded rooms and most of these have balconies overlooking the garden.

The dormitories and day rooms have large open fireplaces, and in addition to these there are hot-water ventilating radiators. Similar radiators are placed in the single-bedded rooms and in the corridors.

The windows are of the sash principle, having large fan-lights over them, and for a hospital of this kind there is probably no better form of window.

In addition to the ventilating radiators there is an elaborate system of inlet and extraction ventilation, so that it would seem as if everything had been done to counteract the drawbacks inherent to a cramped site.

There is an out-patients' department having a separate entrance from Roscoe Street.

The style of architecture is Georgian, and it is carried out in red brick and salmon-coloured terra cotta.

The hospital will accommodate thirty patients and a full staff of nurses and domestic servants.

The architects were Messrs. Gregson and Ould, of Liverpool; the contractors were Messrs. Gerard and Sons, of Swinton; and the clerk of the works was Mr. R. Hardman.

## Figures and Tables

**Figure f1:**
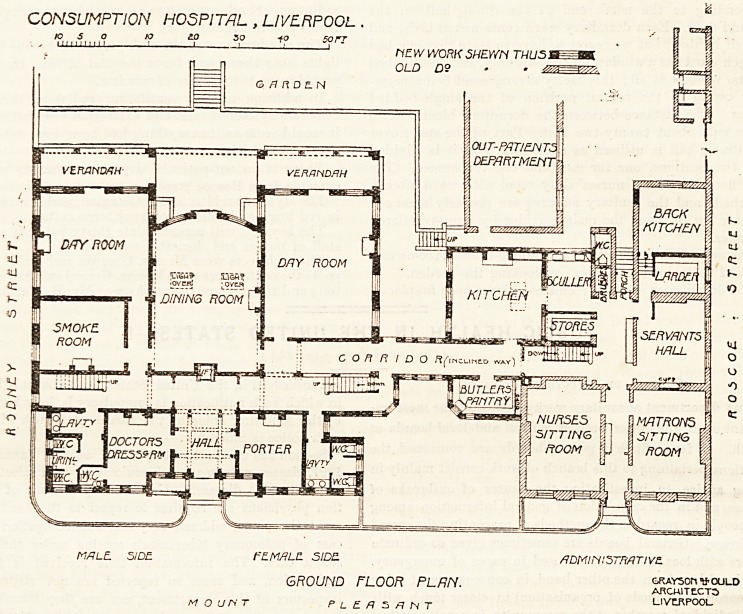


**Figure f2:**